# Differential regulation of muscle protein turnover in response to emphysema and acute pulmonary inflammation

**DOI:** 10.1186/s12931-017-0531-z

**Published:** 2017-05-02

**Authors:** Judith J. M. Ceelen, Annemie M. W. J. Schols, Stefan J. van Hoof, Chiel C. de Theije, Frank Verhaegen, Ramon C. J. Langen

**Affiliations:** 1 Department of Respiratory Medicine, Maastricht University Medical Center, PO box 5800, 6202 AZ Maastricht, The Netherlands; 2 Department of Radiation Oncology (MaastRO), Maastricht University Medical Center, Maastricht, The Netherlands

**Keywords:** Inflammation-induced atrophy, ALP, UPS, Protein synthesis signaling

## Abstract

**Background:**

Exacerbations in COPD are often accompanied by pulmonary and systemic inflammation, and associated with increased susceptibility to and prevalence of weight loss and muscle wasting. Muscle mass loss during disease exacerbations may contribute to emphysema-associated muscle atrophy. However, whether pulmonary inflammation in presence of emphysema differentially affects skeletal muscle, including protein synthesis and degradation signaling pathways has not previously been addressed. The aims of this study were to 1) develop a mouse model of disease exacerbation-associated muscle wasting, 2) evaluate whether emphysema and muscle wasting can be monitored non-invasively and 3) assess alterations in muscle protein turnover regulation.

**Methods:**

Emphysema was induced by three, weekly intra-tracheal (IT) elastase (E) or vehicle control (vc) instillations, followed by one single IT-LPS bolus (L) or vc instillation to mimic pulmonary inflammation-driven disease exacerbation. Consequently, four experimental groups were defined: vc/vc (‘C’), E/vc (‘E’), vc/LPS (‘L’), E/LPS (‘E + L’). Using micro cone-beam CT-scans, emphysema development and muscle mass changes were monitored, and correlated to muscle weight 48 h after LPS instillation. Protein turnover signaling was assessed in muscle tissue collected 24 h post LPS instillation.

**Results:**

Micro-CT imaging correlated strongly with established invasive measurements of emphysema and muscle atrophy. Pulmonary inflammation following LPS instillation developed irrespective of emphysema and body and muscle weight were similarly reduced in the ‘L’ and ‘E + L’ groups. Accordingly, mRNA and protein expression levels of genes of the ubiquitin-proteasome pathway (UPS) and the autophagy-lysosomal pathway (ALP) were upregulated in skeletal muscle following IT-LPS (‘L’ and ‘E + L’). In contrast, mTOR signaling, which controls ALP and protein synthesis, was reduced by pulmonary inflammation (‘L’ and ‘E + L’) as well as emphysema as a single insult (‘E’) compared to control.

**Conclusion:**

Changes in lung tissue density and muscle mass can be monitored non-invasively to evaluate emphysema and muscle atrophy longitudinally. Acute loss of muscle mass evoked by pulmonary inflammation is similar in control and emphysematous mice. Although muscle atrophy cues in response to pulmonary inflammation are not altered by emphysema, emphysema itself affects protein synthesis and ALP signaling, which may interfere with muscle mass recovery and impair maintenance of muscle mass in emphysema.

**Electronic supplementary material:**

The online version of this article (doi:10.1186/s12931-017-0531-z) contains supplementary material, which is available to authorized users.

## Background

Chronic obstructive pulmonary disease (COPD) is characterized by persistent airflow obstruction, resulting from inflammation and remodeling of the airways and, in some patients may include development of emphysema [1]. A major extra-pulmonary hallmark of COPD is muscle wasting which has been shown to be an independent predictor of exercise performance [2] and of mortality [3]. Moreover, COPD patients with an emphysematous phenotype appear to be more prone to skeletal muscle wasting [4].

Skeletal muscle mass is determined by cellular processes that include proteolysis and synthesis, but their contribution to muscle wasting in emphysematous COPD patients is still unclear [5]. Acute exacerbations in COPD are a temporary worsening of the disease which accelerate disease progression and decrease survival [6]. COPD patients that suffer from frequent exacerbations are more prone to weight loss and muscle wasting [7, 8]. Furthermore, Alahmari et al. showed that frequent exacerbations are associated with increased muscle weakness and a decline in exercise capacity [9].

Exacerbations can be triggered by bacterial respiratory infections [10] and are often accompanied by pulmonary and systemic inflammation [11, 12]. Previous studies have shown that pulmonary inflammation evoked by the bacterial component LPS, is sufficient to induce acute loss of skeletal muscle mass [13, 14]. Furthermore, several studies have shown that inflammation causes a disturbance in muscle protein turnover [15, 16].

However, the effects of underlying emphysema on muscle protein turnover regulation and acute pulmonary inflammation-induced muscle atrophy have not been studied. Elastase-induced emphysema is an interesting experimental model as pulmonary inflammation is absent [17], and no changes in muscle mass have been observed [18]. Conversely, smoke-induced emphysema in mice is accompanied by low-grade inflammation and a slight decrease in muscle mass [19], whereas emphysema and a strong pulmonary and systemic inflammatory response in SP-C/TNF transgenic mice is accompanied by a marked reduction in body and muscle weight. Moreover, impaired muscle regeneration observed in these mice suggests that emphysema and inflammation may impair the recovery of atrophied muscle [20].

We postulate that the cumulative effects of acute loss of muscle mass during disease exacerbations and impaired muscle regrowth during stable disease underlies COPD-associated muscle atrophy.

To discern between the effects of inflammation and emphysema during exacerbation we adopted the model of Kobayashi et al., in which a single intratracheal instillation of LPS evokes exacerbation-like features in mice with elastase-induced emphysema [17].

Our first objective was to evaluate muscle wasting in this model of COPD exacerbation, and secondly to assess emphysema and muscle mass changes non-invasively. Emphysematous changes were assessed by quantifying the percentages of low attenuation area (LAA%) using micro computed tomography (μCT), and validated by comparing to the mean linear intercept (Lm), the current golden standard for measuring emphysema. Muscle volumes were also determined using μCT scan analysis, and validated using the skeletal muscle wet weights. The third objective was to investigate protein turnover regulation, including proteolysis and protein synthesis signaling, associated with muscle mass changes.

## Methods

### Animals and experimental protocol

This study was approved by the Institutional Animal Care Committee of Maastricht University. Twelve-week-old C57BL/6 J male mice (Charles River Laboratories International, Wilmington, MA) were randomly divided into four groups, each subjected to one of the following pre-treatment/treatment regimens: controls receiving only vehicle control (vc) solutions; vc/vc, designated ‘C’), elastase pre-treated followed by vc treatment (E/vc, designated ‘E’), vc pre-treated and LPS-treated (vc/L, designated ‘L’) and both elastase pre-treated followed by LPS-treatment (E/L, designated ‘E + L’). Animals received 3 weekly intratracheal (IT) instillations with 4 U of porcine pancreatic elastase (PPE) (Wako Instruchemie BV, Delfzijl, Netherlands) dissolved in 50 μl sterile PBS, or 50 μl of PBS alone (vc). 21 days after the last elastase instillation, the degree of emphysema was determined by μCT-scan analysis. Then mice received an IT instillation of 2.5 μg LPS per gram mouse (*Escherichia coli*, serotype O55:B5; Sigma, St. Louis, MO) dissolved in 50 μl sterile 0.9% NaCl or 50 μl sterile 0.9% NaCl alone (vc). Mice were sacrificed at 24 h after LPS to assess signaling (*n* = 8–12/group) or 48 h after LPS to assess muscle atrophy (*n* = 11/group) [14]. The lungs were rinsed with cell culture medium (i.e., Dulbecco’s Modified Eagle’s Medium containing 0.5% FBS, 50 U/ml penicillin, and 50 mg/ml streptomycin) to obtain bronchoalveolar lavage fluid (BALf). Alternatively, lungs were isolated and fixed for histological purposes. The soleus, gastrocnemius, tibialis anterior, plantaris, and EDL muscles were collected from both hind limbs, using standardized dissection methods, weighed and stored at − 80 °C for RNA and protein extraction.

### μCT imaging and analysis for non-invasive assessment of emphysema and muscle mass

Mice were anesthetized with a mixture of air and 4% isoflurane and scanned using a cone beam, μCT scanner (XRAD-225Cx, Precision X-Ray, North Branford, USA) at a dose of 0.28 Gy. μCT images were acquired at 80 kVp and reconstructed to a 3D image volume with an isotropic voxel spacing of 0.2 mm. Resulting image data were analyzed using SmART-Plan (version 1.3.6) [21]. A selection of the lungs was made and the density of each voxel was plotted in a density histogram. The LAA threshold was set at −426 Hounsfield units (HU). The LAA% was calculated as the ratio of LAA to the total lung area. To validate μCT evaluation of emphysema, LAA% values were subsequently correlated to histological assessment of airspace enlargement (described below).

The muscle of the hindlimbs was delineated using SmART-Plan. Muscle volume was determined as the total selected volume minus air and bone (thresholds −460 and 520 HU respectively). Muscle volumes were then converted by using CT to mass density calibration function, established using the same imaging protocol. The HU to density calibration was performed using a small phantom with a diameter of 29.5 mm, with known 3.6 mm diameter tissue substitute inserts. The tissue substitute inserts were derived from a clinical radiotherapy phantom (Model 467; Gammex RMI, Middleton, WI). A cone-beam CT scan was acquired of this phantom and loaded into SmART-Plan in which all inserts were delineated. The regions of interest for each insert had a volume of 25 mm^3^. That volume of 25 mm^3^ is smaller than the volume of the inserts, because a margin was used at the edges of the inserts for structure delineation. Average HU values were determined from the regions of interest. Two linear functions were fitted to the data points, the first conversion function covers all HU values up to and including the intercept of the two linear functions, and the second function covers all higher HU values. The parameters of the first linear function fit, for HU values up to 162, of the form, density = A*HU + B were: A = 9.508 * 10^−4^ and B = 0.8405. The parameters for the second function fit, for HU values of 163 and higher, were: HU, A = 2.7191*10^−4^ and B = 0.9506. Density of each voxel was calculated, multiplied with the volume of the voxel and summed, generating the mass of the selected muscle. To validate μCT assessment of muscle mass, these values were subsequently correlated to muscle wet weights.

### Histological assessment of emphysema

The lungs were fixated by infusion of 4% paraformaldehyde through a tracheal cannula according to ATS/ERS guidelines for quantitative assessment of lung structure [22, 23]. After excision, the lung was immersed in fresh fixative for 24 h. The lung lobes were embedded in paraffin, cut into 4 mm transverse sections and stained with haematoxylin and eosin staining for histological analysis.

Enlargement of alveolar spaces was determined by quantifying the mean linear intercept (Lm) using image analysis software (Image J 1.33). Sections were scanned using a dotslide light microscopy slide scanner at 100× magnification (Olympus, Hamburg, Germany) and analyzed entirely. Pictures were converted to black (tissue) and white (background) and cutting artifacts or hilar structures (airway or blood vessel with a diameter larger than 50 μm) were excluded from the analysis. Furthermore, background noise was removed using a threshold of 3 pixels. The Lm was measured over the whole lung section by placing a grid on each pixel line. The mean of all white pixel lines gives the average distance between alveolar surfaces, or the Lm.

### Assessment of lung inflammation

BALfluid was centrifugated (5 min at 1500 rpm, 4 °C), pelleted cells were resuspended in differentiation medium (i.e., Dulbecco’s Modified Eagle’s Medium containing 0.5% FBS, 50 U/ml penicillin, and 50 mg/ml streptomycin) and incubated for 3 h at 37 °C to obtain conditioned medium (CM). CM was centrifugated and the supernatant was stored at −80 °C until further use. NF-κB luc-reporter cells were grown on Matrigel (Becton Dickinson Labware, Bedford, MA) and differentiated into myotubes (as described previously) [24]. Myotubes were incubated with CM for 6 h at 37 °C, whereafter they were harvested in Reporter lysis buffer (Promega, Madison, USA). Luminescence was determined using a luminometer (Berthold Lumat LB 9507, Belgium).

### RNA isolation

RNA was isolated from homogenized gastrocnemius muscle from mice sacrificed 24 h post LPS using the RNeasy plus mini kit (Qiagen). cDNA was made with the Tetro cDNA Synthesis kit (GC biotech). Primer sequences of transcripts of interest are provided in Additional file 1: Table S1. The relative DNA starting quantities of the samples were derived using LinRegPCR software (Version 2014.0, Ruijter). The expression of genes of interest was normalized to the geometric average of four reference genes (cyclophilin A, beta-2-microglobulin, 18S, RPL13a) by the GeNorm software.

### Western blotting

Gastrocnemius muscle from mice sacrificed 24 h post LPS was processed and analyzed as previously described [25]. In short, muscle was ground to powder using an N_2_-cooled steel mortar. The powder (~20 mg) was lysed in IP-lysis buffer containing protease inhibitors (Complete; Roche Nederland, Woerden, Netherlands), using a rotating blade tissue homogenizer (Polytron homogenizer, Kinematica). Total protein concentration of the supernatant was determined with a BCA protein assay kit (Pierce Biotechnology, #23225, Rockford, IL) according to manufacturer’s instructions. Laemmli buffer was added and samples were denatured by heating at 100 °C for 5 min. Samples were analyzed by western blot. Briefly, 10 μg of protein per lane were separated on a CriterionTM XT Precast 4–12% or 12% Bis-Tris gel (Bio-Rad Laboratories, Veenendaal, Netherlands) and transferred to a nitrocellulose transfer membrane (Bio-Rad Laboratories) by electroblotting. The membrane was stained with Ponceau *S* solution (0.2% Ponceau S in 1% acetic acid; Sigma-Aldrich Chemie) to control for protein loading. The membranes were blocked, washed in TBS-Tween^0.05%^ and incubated overnight at 4 °C with primary antibodies. A list of used antibodies is provided in Additional file 1: Table S2. All antibodies were diluted 1/1000 in TBS-Tween^0.05^. Blots were then washed and probed with a horseradish peroxidase-conjugated secondary antibody (Vector Laboratories, Burlingame, CA) and visualized with chemiluminescence (Supersignal West Pico or Femto Chemiluminescent Substrate; Pierce Biotechnology) in a LAS-3000 Luminescent Image analyzer (Fujifilm, Tokyo, Japan). Bands were quantified using the Quantity One software (Bio-Rad, version 4.5.0). All data were corrected for protein loading as determined after Ponceau S staining.

### Statistical analysis

Data are shown as means ± SE. Comparisons were computed using SPSS version 22.0. For assessment of significance between groups and genotypes an independent samples *T*-test was used. A *p* value <0.05 was considered statistically significant. Asterisks above a bar refer to a comparison with the respective control (L vs. C and E + L vs. E). An asterisk above a line refers to a comparison between E vs. C or E + L vs. L. 0.05 < *p* < 0.1 was considered a trend.

## Results

### Assessment and validation of emphysema in mice using μCT

**Fig. 1:**
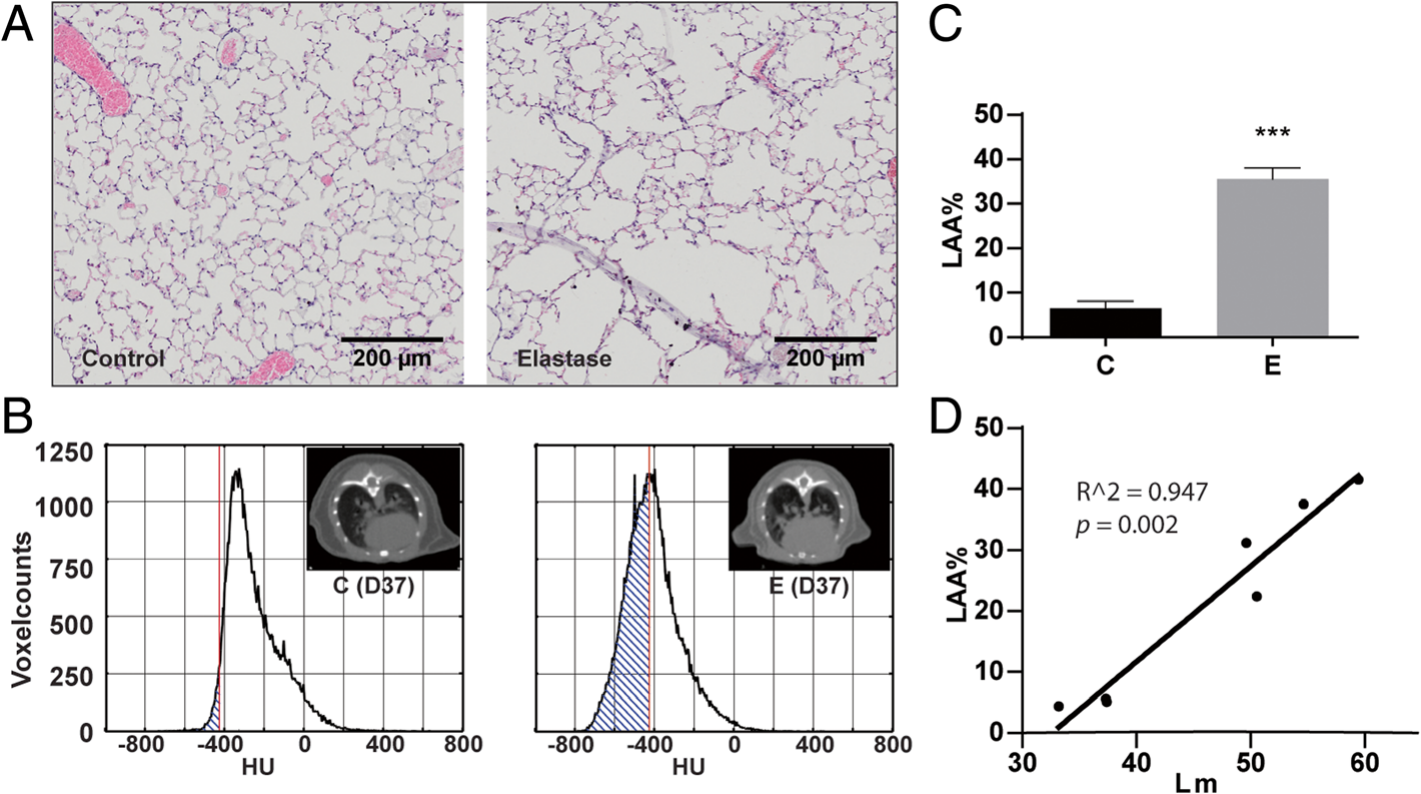
Development of emphysema after instillation with elastase. Mice were intratracheally instilled with elastase to induce emphysema. **a** lungs were isolated 48 h after IT-LPS/NaCl and sections were stained with H&E. **b** μCT scans were analyzed creating a density histogram of the voxels in the selected lungs. The LAA threshold was set to −426 HU. The LAA% was calculated using the ratio of the total LAA volume (shaded area) to the total lung volume. The *upper right* corner shows a representative μCT image. **c** Changes in LAA% after IT-elastase, as determined by μCT scan analysis. *n* = 46 per group. **d** Correlation between μCT-derived LAA% and the Lm (both determined 48 h after NaCl or LPS), *n* = 7. *** *p* < 0.001

Pulmonary emphysema is characterized by the destruction of alveolar walls, leading to enlargement of alveolar spaces [1]. To verify development of emphysema in response to intratracheal elastase, lungs were isolated and lung sections were stained. Alveolar spaces were considerably enlarged in response to elastase (Fig. 1a). In order to determine development of emphysema non-invasively in vivo, μCT scans were made and analyzed, and the percentage of low attenuation area (LAA%) was calculated for each lung (Fig. 1b). Mice that received elastase had a significantly higher LAA% than the control mice (Fig. 1c). To verify this method, emphysema was also assessed as airspace enlargement by histological determination of the mean linear intercept (Lm) in a subset of lungs. LAA% and Lm highly correlated (Fig. 1d).

To determine whether emphysema affected pulmonary inflammation in response to IT-LPS, NF-κB luc-reporter cells were incubated with medium conditioned by cells obtained from BALfluid. No differences were observed in the basal inflammatory state, and the increase in luciferase activity indicated a similar extent of pulmonary inflammation in control and emphysematous mice post IT-LPS (Additional file 2: Figure S1).

### Skeletal muscle atrophy in response to pulmonary inflammation in control and emphysematous mice

**Fig. 2:**
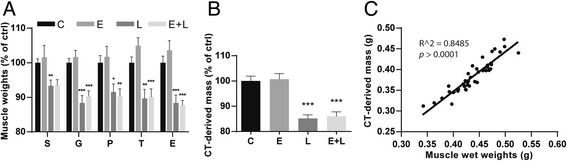
Pulmonary inflammation causes muscle atrophy in control and emphysematous mice. Mice were intratracheally instilled with elastase to induce emphysema or vc, followed by a single bolus of LPS or vc. **a** 48 h after LPS, mice were sacrificed and skeletal muscle wet weights were measured and corrected for tibia length. **b** 48 h after LPS or vc, μCT scans were made and muscle mass was determined. **c** Correlation between μCT-derived muscle mass and combined muscle wet weights. * *p* < 0.05, ** *p* < 0.01, *** *p* < 0.001. *n* = 11–23 per group

After LPS, a rapid decrease in body- (Additional file 3: Figure S2) and skeletal muscle wet weights (Fig. 2a) was observed for control and emphysematous mice. Prior to collection of tissues, μCT scans were made 48 h after LPS and muscle volume was determined to evaluate whether changes in muscle mass can be measured non-invasively (Fig. 2b). A similar magnitude of decrease of 10–15% was observed in μCT-derived muscle mass (Fig. 2b) compared to muscle wet weights (Fig. 2a). Moreover, μCT-derived muscle mass and combined muscle wet weights highly correlated (Fig. 2c).

### Alterations in protein turnover signaling in muscle of emphysematous mice with and without pulmonary inflammation

**Fig. 3:**
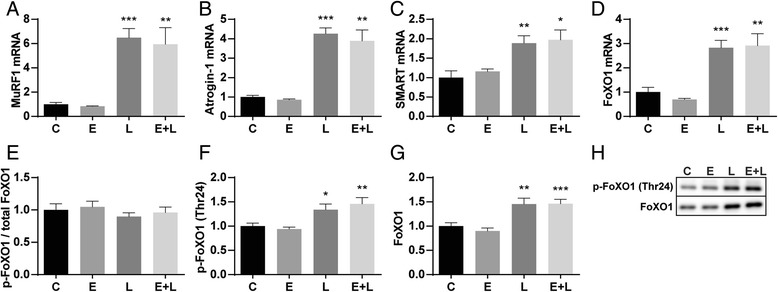
Activation of the UPS in skeletal muscle in response to pulmonary inflammation. Mice were intratracheally instilled with elastase and LPS or respective vc. At 24 h after LPS gastrocnemius muscle was collected for evaluation of mRNA abundance of (**a**) MuRF1, (**b**) Atrogin-1, (**c**) SMART and (**d**) FoXO1, normalized to GeNorm and expressed as fold change compared to control. Alternatively, protein levels of (**f**) phosphorylated FoXO1 (Thr24) and (**g**) total FoXO1 were assessed. **h** Representative western blots of indicated proteins. **e** Ratio of phosphorylated FoXO1 over total FoXO1. *n* = 8–12 per group. * *p* < 0.05, ** *p* < 0.01, *** *p* < 0.001

Activation and involvement of effectors of UPS-mediated proteolysis in pulmonary inflammation-induced muscle atrophy has been demonstrated previously [13, 14]. mRNA transcript levels of the E3 ubiquitin ligases MuRF1, Atrogin-1 and SMART were significantly increased after LPS compared to vc instillation in both control as well as emphysematous mice (Fig. 3a-c), indicating activation of upstream UPS signaling by pulmonary inflammation. The expression of Atrogin-1 and MuRF1 is regulated by activation of FoXO family of transcription factors. Phosphorylation on Thr24 by Akt inactivates FoXO1 resulting in nuclear export and inhibition of transcriptional activity [26]. Although the FoXO1 p/total ratio did not change (Fig. 3e), levels of phosphorylated FoXO1 were increased (Fig. 3f), and accompanied by a similar increase in total FoXO1 protein abundance (Fig. 3g). mRNA transcript levels of FoXO1 were significantly increased (Fig. 3d), suggesting increased de novo FoXO1 protein synthesis.

**Fig. 4:**
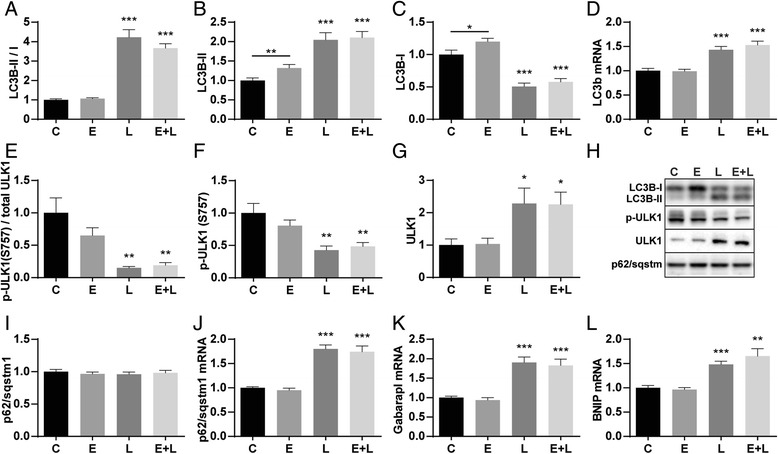
Activation of the ALP in response to pulmonary inflammation. Mice were intratracheally instilled with elastase and LPS or respective vc. Protein levels of (**b**) LC3B-II, (**c**) LC3B-I, (**f**) phosphorylated ULK1 (ser757), (**g**) total ULK1 and (**i**) p62 were assessed in lysates of gastrocnemius muscle tissue with western blot analysis. **h** Representative western blots of indicated proteins. Ratio of (**a**) LC3B-II over LC3B-I and (**e**) phosphorylated ULK1 over total ULK1. mRNA transcript levels of (**d**) LC3B, (**j**) p62, (**k**) Gabarapl and (**l**) BNIP were determined, normalized to geNorm, and expressed as fold change compared to control. *n* = 8–12 per group. * *p* < 0.05, ** *p* < 0.01, *** *p* < 0.001

Besides UPS, there is a role for ALP in muscle proteolysis in the determination of protein turnover signaling. During active autophagy the cytosolic form of LC3 (LC3B-I) is conjugated to the lipidated form (LC3B-II), resulting in recruitment to the autophagosomal membrane [27]. In both control and emphysematous mouse muscle, the LC3B-II/I ratio was significantly increased (Fig. 4a), which is explained by the decreasing levels of LC3B-I (Fig. 4c) and increasing levels of LC3B-II (Fig. 4b). This was accompanied by increased mRNA levels of LC3B after LPS (Fig. 4d). Interestingly, both LC3B-I as well as LC3B-II levels were significantly higher in emphysematous mice compared to control, although this was not reflected in the ratio. ULK1 ser757 phosphorylation by mTOR prevents autophagosome formation [28]. In both control and emphysematous mouse muscle, the ULK1 ratio was significantly decreased (Fig. 4e), due to decreased levels of phosphorylated ULK1 (Fig. 4f). Total levels of ULK1 were increased after LPS (Fig. 4g), suggesting stabilization of the protein. p62 can bind ubiquitinated proteins and LC3B, thereby targeting the autophagosome and facilitating clearance of ubiquitinated proteins [29]. There was no change in p62 protein levels, however mRNA levels increased significantly after LPS (Fig. 4i-j), suggesting that autophagy-mediated turnover of p62 is increased after LPS. Also mRNA levels of Gabarapl, a ubiquitin-like protein required for the formation of autophagosomal membranes, and BNIP, a membrane-bound receptor that can bind LC3B or Gabarapl, were increased after LPS (Fig. 4k-l).

**Fig. 5:**
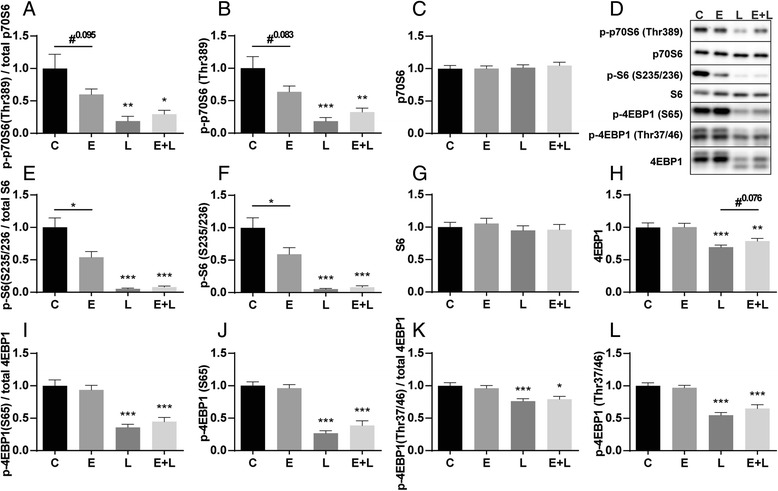
Decreased protein synthesis signaling in response to pulmonary inflammation. Mice were intratracheally instilled with elastase and LPS or respective vc. Protein levels of (**b**) phosphorylated p70S6 (Thr389), (**c**) total p70S6, (**f**) phosphorylated S6 (ser235/236), (**g**) total S6, phosphorylated 4EBP1 (**j**) ser65 and (**l**) Thr37/46 and (**h**) total 4EBP1 were assessed in lysates of gastrocnemius muscle tissue with western blot analysis. (**d**) Representative western blots of indicated proteins. Ratio of (**a**) phosphorylated p70S6 over total p70S6, (**e**) phosphorylated S6 over total S6, (**i**) phosphorylated 4EBP1 (ser65) and (**k**) phosphorylated 4EBP1 (Thr37/46) over total 4EBP1. *n* = 8–12 per group. * *p* < 0.05, ** *p* < 0.01, *** *p* < 0.001, # represents a trend

**Fig. 6:**
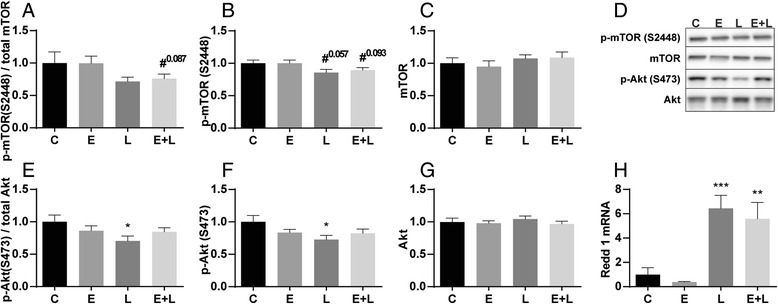
Alterations in REDD1 but not Akt and mTOR phosphorylation levels in response to pulmonary inflammation. Mice were intratracheally instilled with elastase and LPS or respective vc. Protein levels of (**b**) phosphorylated mTOR (ser2448), (**c**) total mTOR, (**f**) phosphorylated Akt (ser473) and (**g**) total Akt were assessed in lysates of gastrocnemius muscle tissue with western blot analysis. **d** Representative western blots of indicated proteins. Ratio of (**a**) phosphorylated mTOR over total mTOR and (**e**) phosphorylated Akt over total Akt. **h** mRNA transcript levels of REDD1 were determined, normalized to geNorm, and expressed as fold change compared to control. *n* = 8–12 per group. * *p* < 0.05, ** *p* < 0.01, *** *p* < 0.001, # represents a trend

Indices of protein synthesis signaling, including the ratio of p70S6, S6 and 4EBP1 decreased significantly after LPS (Fig. 5a, e, i and k), which is explained by a decrease in phosphorylation (Fig. 5b, f, j, l). Interestingly, phosphorylation of S6 and p70S6 (trend only) were already decreased in emphysematous mice compared to control, which is also reflected in the ratio. This suggests that in emphysematous mice, there is already a decrease in protein synthesis signaling independent from pulmonary inflammation. mTOR is phosphorylated at ser2448 via the PI3K/Akt pathway and plays a key role in muscle protein turnover. Apart from a slight trend towards decreased phosphorylation of mTOR after LPS, no changes were observed (Fig. 6a-c). The Akt ratio was decreased in the control mice after LPS (Fig. 6e), which is explained by decreased Akt S473 phosphorylation (Fig. 6f). mTORC1 activity can be regulated by REDD1, which stimulates the inhibitory actions of TSC2 on mTORC1 [30]. mRNA transcript levels of REDD1 were highly increased acutely after LPS instillation (Fig. 6 h), in line with the downregulation of targets downstream of mTOR after LPS.

## Discussion

### Non-invasive detection of emphysema and muscle atrophy

The course of muscle wasting in the emphysematous phenotype of COPD remains unresolved. As demonstrated in this work, the use of μCT scan analysis allows non-invasive monitoring of the development of emphysema and assessment of skeletal muscle mass. Several groups have used non-invasive measurements to determine emphysema [17, 31, 32], and the significant correlation between the LAA% measured by μCT and the Lm shown here further confirms the reliability of μCT as a tool for measuring emphysema. Importantly, in addition to providing an accurate measure for emphysema in vivo, μCT scan analysis showed to be reliable in determining muscle mass non-invasively, considering the high and significant correlation between μCT-derived muscle mass and skeletal muscle wet weights. This will allow longitudinal assessment of muscle atrophy to address the impact of disease exacerbations.

### Skeletal muscle atrophy in response to pulmonary inflammation in the presence of emphysema

Bacterial infections may account for 50% of all exacerbations in COPD [10]. Using LPS to induce an acute pulmonary inflammation in mice with elastase-induced emphysema, emphysema progression and immune cell infiltration have been postulated, similar to COPD patients with exacerbations [17]. Although previous studies have shown that acute lung inflammation is sufficient to induce loss of skeletal muscle mass [13, 14], these concern LPS instillations in healthy mice. Since protease imbalance-induced COPD affects the local immune response [33], pulmonary inflammation may develop differently in the emphysematous lung. However, our data suggests that the pulmonary inflammatory response to LPS is similar in naïve and emphysematous mice. Since emphysema may aggravate the systemic consequences of pulmonary inflammation via e.g., deterioration of gas exchange and subsequent hypoxemia, a synergistic or additive effect of muscle hypoxia [25] and inflammatory signaling on muscle atrophy could be anticipated. However, emphysematous mice loose the same amount of body- and muscle weight as healthy mice after acute pulmonary inflammation. This implies that the mechanistic insights previously acquired with the IT-LPS model in non-emphysematous mice [13, 14] very likely apply to the compound model in its current form as well, although systemic effects of interactions between emphysema and pulmonary inflammation may become apparent when a more moderate inflammatory response is evoked. Conversely, in line with previous research [18], there was no evidence of muscle wasting following elastase-induced emphysema. This contrasts the decreases in body and muscle weights described in models of smoke-induced or chronic inflammation-induced emphysema [19, 20], which may suggest that some degree of pulmonary inflammation is required to induce muscle wasting. Correspondingly, no evidence of lung inflammatory signaling or muscle wasting could be detected in emphysematous mice at baseline. The compound model of disease exacerbation characterized in our study will be useful to discern the effects of and interactions between emphysema and pulmonary inflammation on skeletal muscle wasting and other systemic consequences of COPD.

### Muscle protein turnover signaling in response to pulmonary inflammation

The observed muscle wasting likely involves a contribution of increased proteolysis and decreased protein synthesis signaling, with subtle differences in skeletal muscle of healthy compared to emphysematous mice. Increases in expression of E3 ubiquitin ligases MuRF1, Atrogin-1 and SMART indicate that UPS-mediated proteolysis is activated after pulmonary inflammation. Some evidence of muscle ALP activation in pulmonary inflammation was provided in previous work [14, 34], where the expression of autophagy-related genes including LC3B, Bnip3 and GabarapL1 was significantly upregulated after LPS. Here we demonstrate an increased LC3B-II/I ratio, decreased phosphorylation of ULK1 and increased GabarapL, Bnip and p62 mRNA levels, demonstrating similar activation of ALP in skeletal muscle of emphysematous and control mice after pulmonary inflammation.

In emphysematous mice, phosphorylation of p70S6 and S6, as well as p-4EBP1 decreases acutely after LPS similar to control mice with acute pulmonary inflammation. This downregulation of protein synthesis signaling coincides with the downregulation of ULK1 Ser757 phosphorylation, implying that coordination of autophagy initiation and inhibition of protein synthesis during pulmonary inflammation occurs at the level of reduced mTOR activity.

### Alterations in muscle protein turnover signaling in presence of emphysema

Increased muscle proteolysis as the basis of muscle wasting in COPD has been suggested in a number of reports. However, this interpretation was either based on indirect measurements [35] or signaling [36] of proteolysis, or did not differentiate between the emphysematous and other phenotypes. Conversely, reduced muscle and whole body protein synthesis was suggested by direct measurements in emphysema patients [37]. In line with this, our current findings reveal impaired protein synthesis signaling based on the decreased phosphorylation levels of p70S6 and S6 in skeletal muscle of emphysematous mice at baseline. Interestingly, phosphorylation of ULK1 was also slightly decreased in emphysematous mice, suggesting reduced mTOR activity independent of Akt-mTOR signaling as levels of phosphorylated Akt (S473) or mTOR (S2448) did not show any differences. Moreover, significantly increased levels of LC3B-I and –II, accompanied by unaltered LC3B mRNA levels, are indicative of decreased LC3B-I to –II conversion and decreased breakdown of LC3B-II, and alterations in autophagy. These data suggest that protein synthesis signaling as well as ALP regulation is already affected in emphysematous mice.

Differences in protein synthesis signaling may contribute to depressed muscle protein synthesis at baseline [37] or in response to anabolic triggers including exercise as was previously demonstrated [38] in patients with emphysema. Although this does not affect muscle mass in emphysematous mice in the timeframe assessed here, this may ultimately culminate in muscle atrophy when sustained for longer periods or as a consequence of an impaired capacity to recover from loss of muscle mass [39].

## Conclusion

In conclusion, micro-CT scan analysis is a reliable method for non-invasive assessment of emphysema and changes in muscle mass, allowing longitudinal monitoring in mice. Skeletal muscle atrophy and changes in muscle protein turnover signaling as systemic consequences of pulmonary inflammation are not altered in emphysematous mice.

## Additional files

Additional file 1: Table S1. Sequences of primers used for RT-qPCR to assess expression of the indicated genes. **Table S2.** Antibodies used for western blot. (DOCX 17 kb)

Additional file 2: Figure S1. Similar pulmonary inflammation in control and emphysematous mice following IT-LPS instillation. Mice were intra-tracheally instilled with elastase to induce emphysema or vc, followed by a single bolus of LPS or vc. Lungs were lavaged to obtain BALfluid (*n* = 11 or 12/group), and cells isolated from the BALf were used to produce conditioned medium. NF-κB luciferase activity was measured in lysates prepared from C2C12 myotubes after stimulation with conditioned medium. (TIF 774 kb)

Additional file 3: Figure S2. Similar loss of bodyweight in control and emphysematous mice following IT-LPS instillation. Mice were intra-tracheally instilled with elastase to induce emphysema or vc, followed by a single bolus of LPS or vc. Changes in body weight (0 h *n* = 21–23, 24 h *n* = 21–23, 48 h *n* = 10 or 11) after IT-LPS were measured and expressed as a percentage of their respective IT-NaCl time control. (TIF 1284 kb)

## Abbreviations

μCT: Micro computed tomography
ALP: Autophagy-lysosomal pathway
BALf: Bronchoalveolar lavage fluid
C: Control
CM: Conditioned medium
COPD: Chronic obstructive pulmonary disease
E: Elastase-treated
E + L: Elastase- and LPS-treated
HU: Hounsfield units
IT: Intra-tracheal
L: LPS-treated
LAA%: Percentage of low attenuation area
Lm: Mean linear intercept
PPE: Porcine pancreatic elastase
UPS: Ubiquitin-proteasome pathway
vc: Vehicle control
