# Supplementary material for: Differential regulation of muscle protein turnover in response to emphysema and acute pulmonary inflammation
Source: Respir Res. 2017 May 2;18:75. doi: 10.1186/s12931-017-0531-z (PMC5414227; doi:10.1186/s12931-017-0531-z)
Supplement: Supplementary file 1 — Sequences of primers used for RT-qPCR to assess expression of the indicated genes. Table S2. Antibodies used for western blot. (DOCX 17 kb) [file 12931_2017_531_MOESM1_ESM.docx]

Table S1: Sequences of primers used for RT-qPCR to assess expression of the indicated genes

| Gene | Forward primer (5’ to 3’) | Reverse primer (5’ to 3’) |
| --- | --- | --- |
| Cyclophilin A | TTCCTCCTTTCACAGAATTATTCCA | CCGCCAGTGCCATTATGG |
| Beta-2-microglobulin | CTTTCTGGTGCTTGTCTCACTGA | GTATGTTCGGCTTCCCATTCTC |
| 18S | AGTTAGCATGCCAGAGTCTCG | TGCATGGCCGTTCTTAGTTG |
| RPLP0 | GGACCCGAGAAGACCTCCTT | GCACATCACTCAGAATTTCAATGG |
| MuRF1 | CTTCCTCTCAAGTGCCAAGCA | GTGTTCTAAGTCCAGAGTAAAGTAGTCCAT |
| Atrogin-1 | CAGCAGCTGAATAGCATCCAGAT | TCTGCATGATGTTCAGTTGTAAGC |
| SMART | CACAGGGATGTCTGCTACTC | ACACAGTTGTAGCCGTACCTC |
| FoXO1 | AAGAGCGTGCCCTACTTCAAGGATA | CCATGGACGCAGCTCTTCTC |
| LC3B | GAGCAGCACCCCACCAAGAT | CGTGGTCAGGCACCAGGAA |
| p62/SQSTM1 | GAATGTGGGGGAGAGTGTGG | TCTTCTGTGCCTGTGCTGGA |
| Gabarapl | CCCTCCCACCAGTGCTACCAT | TCATCACTGTAGGCCACATACAGAAAA |
| Bnip3 | AGGTTTTCCTTCCATCTCTGTTACTG | TGTGTGAACAGAAGTCAGATCCAAA |
| REDD1 | TCGGCGCTTCACTACTGACC | CCTAACACCCACCCCATTCC |

Table S2: Antibodies used for western blot

| Antibody | Cat. number | Antibody | Cat. number |
| --- | --- | --- | --- |
| p-FoXO1 (Thr24) | #9464 | p-S6 (S235/236) | #4856 |
| FoXO1 | #2880 | S6 | #2217 |
| LC3B | #2775 | p-p70S6 (Thr389) | #9205 |
| p-ULK1 (S757) | #6888 | p70S6 | #9202 |
| ULK1 | #8054 | p-mTOR (S2448) | #2971 |
| p62/sqstm | #5114 | mTOR | #2983 |
| p-4EBP1 (S65) | #9451 | p-Akt (S473) | #9271 |
| p-4EBP1 (Thr37/46) | #9459 | Akt | #9272 |
| 4EBP1 | #9452 |  |  |
