# Supplementary figures and images for: Differential regulation of muscle protein turnover in response to emphysema and acute pulmonary inflammation
Source: Respir Res. 2017 May 2;18:75. doi: 10.1186/s12931-017-0531-z (PMC5414227; doi:10.1186/s12931-017-0531-z)

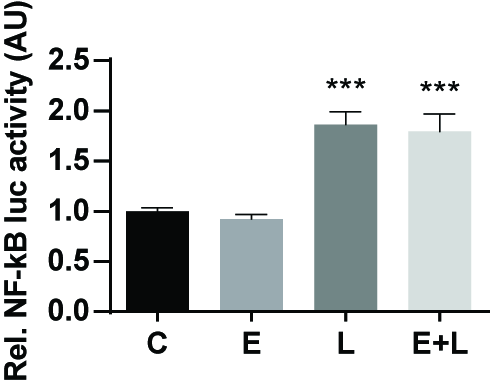

Supplement: Supplementary file 2 — Similar pulmonary inflammation in control and emphysematous mice following IT-LPS instillation. Mice were intra-tracheally instilled with elastase to induce emphysema or vc, followed by a single bolus of LPS or vc. Lungs were lavaged to obtain BALfluid (n = 11 or 12/group), and cells isolated from the BALf were used to produce conditioned medium. NF-κB luciferase activity was measured in lysates prepared from C2C12 myotubes after stimulation with conditioned medium. (TIF 774 kb) [file 12931_2017_531_MOESM2_ESM.tif]

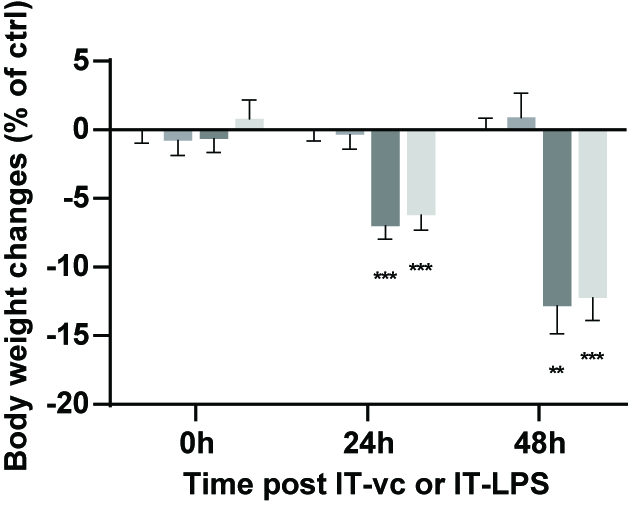

Supplement: Supplementary file 3 — Similar loss of bodyweight in control and emphysematous mice following IT-LPS instillation. Mice were intra-tracheally instilled with elastase to induce emphysema or vc, followed by a single bolus of LPS or vc. Changes in body weight (0 h n = 21–23, 24 h n = 21–23, 48 h n = 10 or 11) after IT-LPS were measured and expressed as a percentage of their respective IT-NaCl time control. (TIF 1284 kb) [file 12931_2017_531_MOESM3_ESM.tif]
